# Retraction: Enhanced Anti-Tumor Effect of Zoledronic Acid Combined with Temozolomide against Human Malignant Glioma Cell Expressing O^6^-Methylguanine DNA Methyltransferase

**DOI:** 10.1371/journal.pone.0268315

**Published:** 2022-05-05

**Authors:** The PLOS ONE Editors

After this article [1] was published, concerns were raised about the mouse tumor sizes reported in Fig 6.

The chart in Fig 6B of the article appears to report tumor sizes of up to 5000 mm^3^, with a standard error of almost 2000 mm^3^ for the no treatment group. In response to queries about these experiments, the corresponding author stated that the y-axis in the tumor size chart in Fig 6B (left panel) is incorrect and should show a maximum of 3500 mm^3^ instead of 7000 mm^3^. The corresponding author provided a revised tumor size chart and the underlying group mean and standard deviation data in which the mean tumor volume for the control group was reported as 2559 mm^3^ ± 873 mm^3^. The corresponding author noted that during the experiment, all efforts were made to minimize suffering, and that, according to the study protocol, the mice were checked once a week and euthanized when the tumor diameter reached 18 mm, the tumor impeded mobility, or the mouse was in poor condition. It was further explained that monitoring of the animals included assessment of eating and drinking; behaviour, posture, breathing, and squeaking; and appearance and condition. A copy of the ethics approval letter for the study was provided. The corresponding author stated that the underlying individual-level tumor volume and mouse weight data are not available.

The *PLOS ONE* Editors consulted with an expert in animal research methodology who assessed the article and the authors’ comments and confirmed that the tumor sizes reported in this article appear to exceed community standards for humane endpoint limits in mouse tumor studies. They also noted that the revised tumor volume data and the information about humane endpoint criteria provided by the corresponding author in follow-up discussion do not appear to be consistent with the tumors shown in the photographs of the mice in Fig 6A.

In light of the above concerns, the *PLOS ONE* Editors retract this article. The editors regret that these concerns were not addressed at the time of the original review process.

JF and NN agreed with retraction. FK did not directly comment on the retraction decision.
